# Chronic endogenous fungal endophthalmitis: diagnostic and treatment challenges: A case report

**DOI:** 10.1097/MD.0000000000025459

**Published:** 2021-04-09

**Authors:** Dian Nadia Abu Talib, Meng Hsien Yong, Rona Asnida Nasaruddin, Jemaima Che-Hamzah, Mae-Lynn Catherine Bastion

**Affiliations:** Department of Ophthalmology, Faculty of Medicine, Universiti Kebangsaan Malaysia, Malaysia.

**Keywords:** antifungal, candida, endogenous fungal endophthalmitis, voriconazole

## Abstract

**Rationale:**

Endogenous fungal endophthalmitis (EFE) is a sight-threatening complication of systemic fungemia. As the prevalence rises, treatment remains a challenge especially when there is a failure in first-line treatment or drug-resistant fungus. This case report studies a case of chronic EFE, focusing on the diagnostic procedures, treatment options, monitoring parameters and the treatment outcome

**Patient concerns:**

A 64-year-old man with underlying well controlled diabetes mellitus was treated with 2 weeks’ course of intravenous antifungal fluconazole for pyelonephritis as his blood culture grew *Candida albicans*. Concurrently, he complained of 3 months of bilateral painless progressive blurring of vision. At presentation, his visual acuity (VA) was light perception both eyes. Ocular examination revealed non granulomatous inflammation with dense vitritis of both eyes.

**Diagnosis:**

He was diagnosed with EFE but the condition responded poorly with the medications.

**Interventions:**

He was treated with intravitreal (IVT) amphotericin B and fluconazole was continued. Vitrectomy was performed and intraoperative findings included bilateral fungal balls in the vitreous and retina with foveal traction in the left eye. Postoperatively, vision acuity was 6/24, N8 right eye and 2/60, N unable for left eye with extensive left macular scar and hole. Vitreous cultures were negative. He received multiple IVT amphotericin B and was started on topical steroid eye drops for persistent panuveitis with systemic fluconazole. Ocular improvement was seen after switching to IVT and topical voriconazole. Despite this, his ocular condition deteriorated and he developed neovascular glaucoma requiring 3 topical antiglaucoma agents. Panretinal photocoagulation was subsequently performed.

**Outcomes:**

At 3 months’ follow-up, his vision acuity remained at 6/24 for right eye and 2/60 for the left eye. There was no recurrence of inflammation or infection in both eyes.

**Lessons:**

Voriconazole could serve as a promising broad spectrum tri-azole agent in cases of failure in first-line treatment or drug-resistant fungus.

## 1 Introduction

*Fungi* are ubiquitous eukaryotic organisms. The 3 important classes which are *molds, yeasts*, and biphasic *fungi* are important ocular pathogens and responsible for many cases of endophthalmitis. The most common cause of endogenous *fungal* endophthalmitis (EFE) is *Candida* species followed by *Aspergillus* species.^[1]^*Candida albicans* are commensal organisms that reside in the human body and are found normally in the gastrointestinal, genitourinary and respiratory tracts.

The unique treatment challenges for endogenous endophthalmitis occur due to its diagnostic difficulty and limited therapeutic options. The yield of positive cultures from vitreous sample is usually low at around 38%.^[2]^

Treatment poses a challenge as most drugs are fungistatic, have limited spectrum activity, and have poor ocular penetration and the growing number of fluconazole resistant species.

## 2 Case presentation

**Figure 1:**
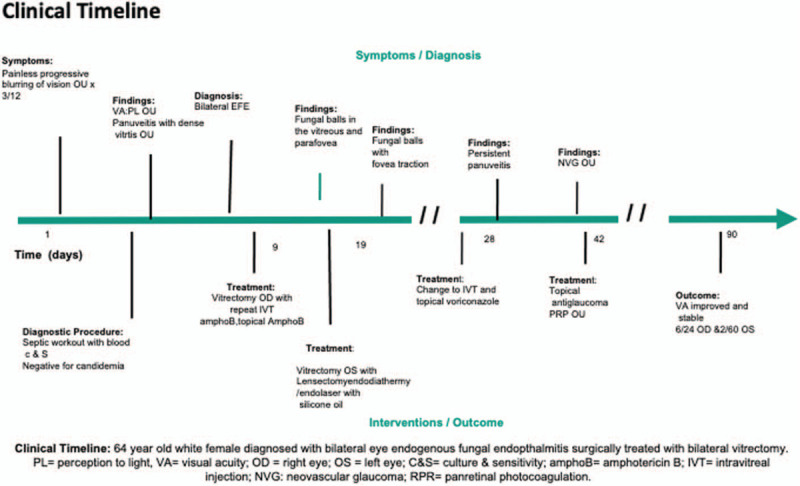
Clinical timeline.

A 64-year-old gentleman with underlying well controlled type-2 diabetes mellitus presented with painless progressive blurring of vision on both eyes for the past 3 months (Fig. 1). He had a history of pyelonephritis with Candidemia at the same time of the onset of his ocular symptoms. The systemic infection was treated with intravenous fluconazole for 2 weeks. He was on single oral hypoglycemic agent with fairly good control of his underlying diabetes that evidenced by his HbA1c level of 6.6% during the admission.

**Figure 2:**
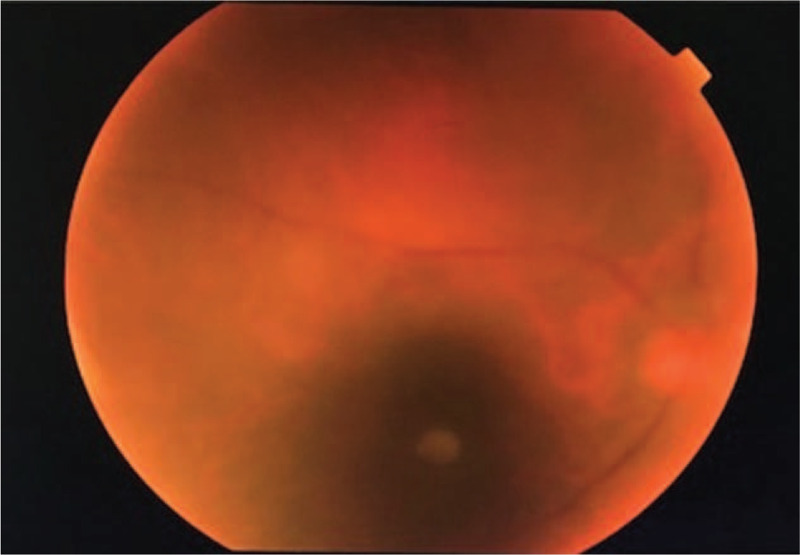
Fundus photo of right eye pre-operation showed dense grade 3 vitritis and silhouette of a fungal ball at the macula.

**Figure 3:**
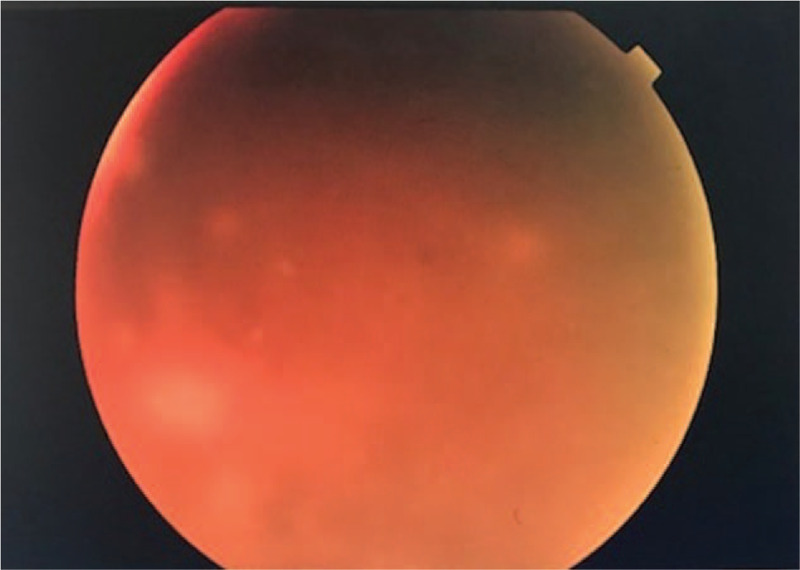
Fundus photo of left eye pre-operation showed dense grade 4 vitritis.

**Figure 4:**
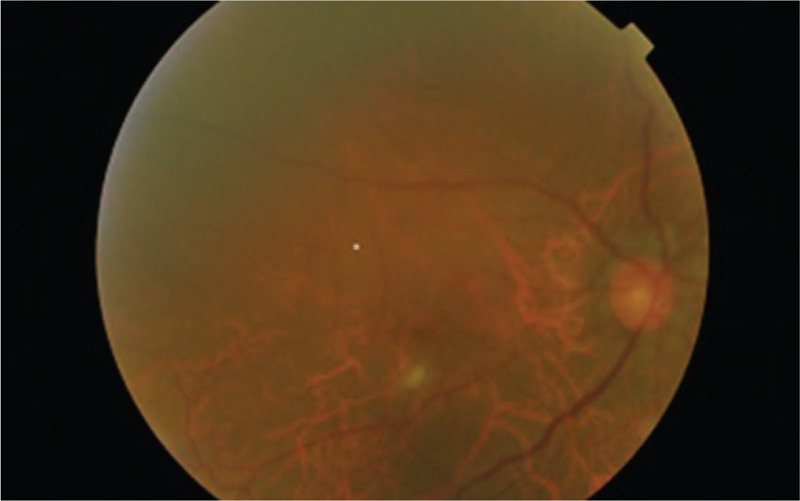
Fundus photo of the right eye post-operation showed the vitreous was more clearer with residual fungal ball at macula.

**Figure 5:**
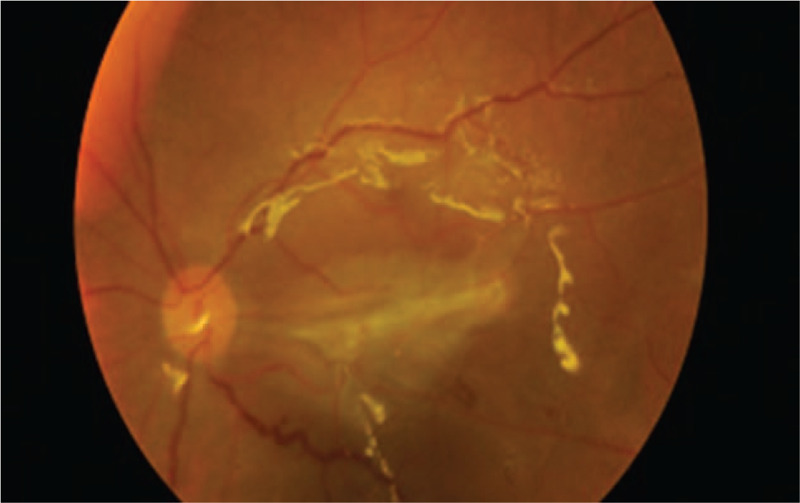
Fundus photo of the left eye post-operation showed extensive left macular scar with silicone oil filled.

On his initial presentation to eye clinic, ocular examination revealed bilateral vision of perception to light. Anterior segment showed conjunctival injection, pigmented keratic precipitates on the inferior part of cornea, and grade 3+ anterior chamber reaction without hypopyon. The iris was normal without nodule or neovascularization. Intraocular pressure was normal on presentation. Fundus revealed hazy view with dense vitritis (Figs. 2 and 3). Brightness-scan showed bilateral vitreous opacities with loculations but flat retina. He was diagnosed as bilateral EFE. Intravitreal (IVT) amphotericin B and intensive topical amphotericin B was started but the condition responded poorly with the medications. He underwent bilateral pars plana vitrectomy with IVT amphotericin B, ceftazidime, and vancomycin of right eye on day 9 and left eye on day-19 of presentation. The left eye operation was including lensectomy, membrane peeling, endolaser, ando pupillary iridotomy, and tamponade with silicone oil 5000 centistokes. Intraoperative findings revealed the presence of strongly adherent fungal balls at parafoveal area of right eye. The left eye showed multiple fungal balls at peripapillary and at the fovea with surrounding traction. Efforts to remove the fungal balls intraoperatively resulted in iatrogenic macula hole in the left eye. Postoperatively at 1 month his best vision acuity was 6/24 for right eye and 2/60 for the left eye (Figs. 4 and 5). Despite the combination of surgical clearance of fungal load, intravenous fluconazole, repeated IVT and intensive topical amphotericin B with topical steroid, his eyes showed persistent panuveitis. At that point of time, differentials of fluconazole-resistant ocular Candidiasis were considered. However, multiple samples of IVT tapping and intraoperative vitreous biopsy obtained were negative for bacterial culture, fungal culture, and cytology. We switched both topical and IVT antifungal to voriconazole 1 week after the pars plana vitrectomy. After the switching, there was improvement with reduction in both anterior chamber reaction and vitritis. Unfortunately, upon follow-up at day 40 patient developed bilateral neovascular glaucoma (NVG) with intraocular pressure of 30 mmHg requiring 3 topical antiglaucoma agents. Fundus fluorescein angiography showed patchy capillary fall-out areas but no leakage. He received and completed few sessions of pan-retinal photocoagulation. Subsequent follow-up up to 6 months revealed stable visual acuity of 6/24 for right eye and 2/60 for left eye and stable intraocular pressure. There was no recurrence of inflammation or infection in both eyes.

## 3 Discussions

### 3.1 Diagnostic challenge

*Candida* EFE is a clinical diagnosis in which there is a progression of *Candida* chorioretinitis with extension into the vitreous forming puff ball like lesions which represent vitreous abscesses. *Candida* chorioretinitis is characterized by the presence of fluffy white creamy lesions at the level of the choroid and retina. The established risk factors include immunosuppressive diseases such as uncontrolled diabetes mellitus, cancer, therapy with broad spectrum antibiotics and immunosuppressive drugs, major surgery especially intra-abdominal surgery, intravenous hyper alimentation, indwelling intravenous catheter, intravenous drug use, as well as neutropenia.

Methods of diagnosis of EFE include both culture and nonculture methods. The extra advantage of using culture method is to be able to get its sensitivity result for antifungal especially in resistant cases, but the drawback is the insensitive yield and long processing time that may cause a delay in treatment. Nonculture methods including antigen detection and molecular techniques allow rapid diagnosis but are more expensive and not widely available. Given the high level of suspicion of fungal endophthalmitis due to history of genitourinary surgery with positive culture of *Candida* in our case, he was treated as EFE despite negative yield from vitreous culture.

### 3.2 Treatment challenge

The approach to treatment of EFE is to treat its source and to achieve adequate concentrations of antifungal agents in the infected tissues. The vascularized compartments which are both choroid and retina are separated from intraocular structures by the blood–retinal barrier. Thus, infection localized to the chorioretinal layers, which are not protected by this barrier, can be treated with systemic antifungal agents. Sight-threatening lesions in the macula and chorioretinitis with vitritis, however, necessitate IVT injection of antifungal agents, with or without vitrectomy.^[3]^

Choices of systemic, IVT, and topical antifungal for treating intraocular candidemia remain a challenge due to the presence of structural barriers and compartmentalisation explained earlier as well as the indolent nature of the infection. For an adequate therapeutic response, both correct drug choice and effective administration are important. Amphotericin B which possesses rapid fungicidal and wide-coverage for various fungi remains the standard therapy of intraocular candidemia despite its variable tissue penetration. This drug can be administered by intraocular routes and systemic but with reported occasional clinical failures.^[4]^ Although fluconazole shows a favorable cerebrospinal fluid and brain penetration which can be extended to ocular structures, its activity may be limited by its fungistatic activity, as well as the upsurge of resistant strains.^[5]^ Finally voriconazole, a second generation of triazoles, is effective as a systemic or locally injected agent. Clinical, pharmacokinetic, pharmacodynamic, and in vitro data suggest this antifungal agent plays an important role in the treatment of ocular infection.^[6]^ The bioavailability of oral formulation is similar with parenteral form making long-term treatment with voriconazole feasible on an outpatient basis.

In our case in view of the persistent inflammation despite surgical intervention and intensive antifungal with amphotericin B and fluconazole, the patient was treated as amphotericin B and/or fluconazole resistance and the antifungal changed to voriconazole. Patient responded well with the change of antifungal.

Fluconazole- and amphotericin B-resistant *Candida* is known as multidrug resistance and has become an emerging problem. A study by Breit et al reported treatment success in all case series of 5 patients who received voriconazole therapy for *Candida* endophthalmitis. Unfortunately, it is impossible to identify whether voriconazole alone was responsible for resolution of disease in these patients as 3 of the patients had received voriconazole in combination with caspofungin.^[4]^

The role of vitrectomy is to remove organisms from the vitreous cavity so that relatively little systemic medication is required. It helps the diffusion of intravenously administered medications, and relieves vitreoretinal traction. Early vitrectomy within a week of diagnosis has been associated with a significantly reduced risk of retinal detachment in eyes with *Candida* EFE.^[7]^ This case was operated within 11 days apart and the patient had relatively good visual recovery in the right eye but modest visual outcome in fellow eye mainly due to the difference in the extent of macula damage caused by direct fungal ball infection to the foveal region. Cases in which the fovea is spared would be expected to have a better visual prognosis. This highlights the importance of early diagnosis and takes into consideration the extent of infiltrate involved and clinical symptoms before deciding which therapeutic options are best. Behera et al retrospectively analyzed 10 years of experience (2006–2015) in managing fungal endophthalmitis. The study concluded a strong clinical suspicion that translates into early vitrectomy plus IVT antifungal leads to favorable visual and structural outcomes especially in regions where incidence of fungal endophthalmitis is high. A long wait till microbiological confirmation to institute antifungal therapy may result in poorer outcome.^[8]^

Successful treatment of ocular candidiasis was defined as disappearance of active inflammation within the eye and either disappearance or scarification of retinal lesions. Follow-up examinations are routinely performed to evaluate the response to therapy and the development of complications such as tractional retinal detachment or more uncommon complications such as NVG which require further intervention. Prolonged or severe inflammation in the ocular structure will cause disturbance of the balance between local stimulators and inhibitors of microvasculature especially release of angiogenic factors, including v*ascular endothelial growth factor* by inflammatory cells mainly macrophages in chronic inflammation.^[9]^ The NVG in our case occurred after successful treatment of ocular candidiasis with resolving inflammation. The fluorescein angiography of the patient showed patchy capillary fall-out areas with no leaky new vessels or vasculitis changes. He was treated with panretinal photocoagulation for the ischemic component. The patient responded to the laser treatment well with regression of rubeosis and stable intraocular pressure.

## 4 Conclusion

In summary, this case highlighted modest visual outcome can be achieved in EFE with combination of early vitrectomy, systemic and IVT antifungal. Voriconazole should be considered if poor response to fluconazole/amphotericin B is encountered. Retinal detachment and macular lesions at presentation were associated with severe vision loss as well as poor prognosis.

## Author contributions

**Conceptualization:** Yong Meng Hsien, Mae-Lynn Catherine Bastion.

**Data curation:** Dian Nadia Abu Talib.

**Investigation:** Dian Nadia Abu Talib.

**Supervision:** Yong Meng Hsien, Rona Asnida Nasaruddin, Jemaima Che-Hamzah, Mae-Lynn Catherine Bastion.

**Validation:** Meng Hsien Yong, Mae-Lynn Catherine Bastion.

**Writing – original draft:** Dian Nadia Abu Talib, Yong Meng Hsien, Rona Asnida Nasaruddin, Jemaima Che-Hamzah, Mae-Lynn Catherine Bastion.

**Writing – review & editing:** Dian Nadia Abu Talib, Yong Meng Hsien, Rona Asnida Nasaruddin, Jemaima Che-Hamzah, Mae-Lynn Catherine Bastion.

Written informed consent for publication of the patient clinical details and clinical images was obtained.
